# Clinical impact of using a more sensitive troponin assay in patients with acute chest pain

**DOI:** 10.1002/clc.23177

**Published:** 2019-03-29

**Authors:** Guangmei Wang, Jiali Wang, Shuo Wu, Wen Zheng, He Zhang, Jingjing Ma, Jiaqi Zheng, Feng Xu, Yuguo Chen

**Affiliations:** ^1^ Department of Emergency Qilu Hospital, Shandong University Jinan China; ^2^ Chest Pain Center Qilu Hospital, Shandong University Jinan China; ^3^ Key Laboratory of Emergency and Critical Care Medicine of Shandong Province Qilu Hospital, Shandong University Jinan China; ^4^ Key Laboratory of Cardiovascular Remodeling and Function Research, Chinese Ministry of Education and Chinese Ministry of Public Health Qilu Hospital, Shandong University Jinan China

**Keywords:** acute coronary syndrome, chest pain, troponin

## Abstract

**Background:**

More sensitive troponin assays have the potential to better evaluate patients with suspected acute coronary syndrome (ACS). Meanwhile, they may result in avoidable diagnostic testing.

**Hypothesis:**

Our aim was to determine the clinical impact of implementing a more sensitive cardiac troponin I (cTnI) assay in patients with acute non‐traumatic chest pain presenting to the emergency department (ED).

**Methods:**

This is a pre‐post cohort study. A total of 1201 consecutive patients with acute non‐traumatic chest pain or equivalent ischemic symptoms suggestive of ACS were allocated to two groups according to the cTnI assay used. The outcomes included the ED length of stay (LOS), hospital admission rate, the use of procedures and the incidence of major adverse cardiac events (MACE) at 30 days.

**Results:**

The introduction of the more sensitive troponin assay shortened ED LOS (odds ratio [OR] 0.39, 95% confidence interval [CI] 0.28‐0.54) regarding patients discharged home directly, increased the hospital admission rate (OR 1.43, 95% CI 1.12‐1.84), the use of echocardiography (OR 1.58, 95% CI 1.22‐2.06), coronary computed tomography angiography (OR 1.78, 95% CI 1.04‐3.06), coronary angiography (OR 1.53, 95% CI 1.10‐2.12) and percutaneous coronary intervention (OR 2.42, 95% CI 1.58‐3.70) regarding patients discharged or admitted. The incidence of MACE did not decrease significantly (OR 0.61, 95% CI 0.27‐1.37).

**Conclusions:**

The introduction of the more sensitive troponin assay appeared to result in less time spent in the ED regarding patients discharged home directly, but prompted more hospitalizations and procedures without impacting the incidence of MACE.

## 1 INTRODUCTION

Cardiac biomarkers are the mainstay of the evaluation of patients with suspected acute coronary syndrome (ACS). The preferred biomarker is cardiac troponin (cTn), including cardiac troponin I (cTnI) and cardiac troponin T (cTnT). The elevated cTn level is fundamental to the diagnosis of acute myocardial infarction (AMI).^1^, ^2^, ^3^ There are many assays to detect cTn. Due to the improved analytical sensitivity, the new and more sensitive cTn assays can detect myocardial injury substantially earlier with high precision than the previous generation of assays.^4^ These assays adopted a lower threshold for the detection of myocardial injury. However, increased sensitivity is accompanied by decreased specificity, and only a third of patients who reclassified with the high‐sensitivity assay as myocardial injury had a diagnosis of type 1 myocardial infarction (MI).^5^, ^6^ The dynamic changes in high‐sensitivity cTn concentration, quantified as delta cTn has been proposed as a tool for the improvement of specificity.^7^ But the optimal delta cTn criteria for diagnosing AMI were influenced by many uncertainties, such as time between measurements, individual assay, the range of troponin elevation. The usefulness of delta cTn remains elusive.^7^, ^8^, ^9^

Troponin detection exerts a great influence on clinical decision‐making. More sensitive troponin assays have the potential to better evaluate the patients with suspected ACS presenting to the emergency department (ED), allowing for earlier safe discharge of low‐risk chest pain and earlier recognition of AMI.^10^ Conventional troponin assays could lead to an inappropriate discharge, while more sensitive troponin assays may result in avoidable diagnostic testing. Published data about the impact of the use of more sensitive cTn assays compared with the conventional ones on the actual managements and outcomes of chest pain patients presenting to the ED are inconclusive.^6^, ^11^, ^12^, ^13^ More researches are needed to test whether the clinical consequences are altered when the more sensitive cTn assays are used in different health settings.

The introduction of a more sensitive cTnI assay in our laboratory allowed us a unique opportunity to assess the clinical implications of two different cTnI assays. In this study, we decided to investigate whether the alteration of cTn assays was associated with changes in (a) length of stay (LOS) in the ED; (b) hospital admission rate; (c) the use of diagnostic measures and cardiovascular therapies; and (d) the incidence of major adverse cardiac events (MACE) at 30 days.

## 2 METHODS

### 2.1 Study population

This is a pre‐post cohort study. We performed a retrospective analysis of data which were collected prospectively. We identified all consecutive patients aged ≥18 years presenting to the ED with acute non‐traumatic chest pain or equivalent ischemic symptoms suggestive of ACS with an onset or peak within the last 24 hours in Qilu Hospital of Shandong University, a tertiary and teaching hospital with about 110 000 ED visits per year. Equivalent ischemic symptoms suggestive of ACS included shortness of breath, nausea, vomiting, jaw pain, arm pain, etc.^14^

On May 10, 2016, a new Access AccuTnI+3 (Beckman Coulter, Inc. Brea, California) was introduced into our laboratory. Previously, the AccuTnI (Beckman Coulter, Inc. Fullerton, California), a conventional cTnI assay had been used for more than 10 years.

The study cohort was allocated to two groups according to the cTn assay used: (a) study period 1 (from September 1, 2015 to May 9, 2016), using the conventional cTnI assay; (b) study period 2 (from September 1, 2016 to May 9, 2017), using the more sensitive cTnI assay. Patients presenting in the time interval between the two periods were not considered, to alleviate the effects of seasonal variation.

Patients were excluded from analysis if (a) a point‐of‐care assay to test cTnI levels was used, (b) they were transferred from another hospital, (c) there was a new ST‐segment elevation or left bundle branch block in the initial electrocardiogram (ECG) suggesting ST‐segment elevation MI, (d) no cTnI level was measured, and (e) they were unwilling to provide informed consent.

The study was approved by the ethics committee of the hospital, and all patients included provided written informed consent.

### 2.2 Troponin assays

Patients in the study period 1 underwent cTnI testing by Access AccuTnI, which had a 99th percentile upper reference limit (URL) of 0.04 ng/mL with a median imprecision of 14% coefficient of variation (CV), and a 10% CV at 0.06 ng/mL.^15^ Our central laboratory used 0.06 ng/mL suggested by the manufacturer as the diagnostic threshold for this conventional cTnI assay.

Patients in the study period 2, underwent testing using Access AccuTnI+3. The 10% CV was at 40 ng/L. The 99th percentile URL was 30 ng/L, and this was used as the diagnostic threshold for this more sensitive cTnI assay.

### 2.3 Patient management

All patients underwent an initial assessment, including clinical history, physical examination, ECG, and blood tests. Generally, patients who were considered to have a benign cause for their complaint were discharged directly from the ED, while those who had a life‐threatening condition and needed urgent invasive management were admitted to hospital immediately. The remaining patients were scheduled for admission or observation. Blood samples were drawn based on physician judgment, but not at established time intervals.

There was no relevant change in patient flow, staff to patient ratio, cardiac catheterization laboratory availability, or funding practice during the enrollment period. Clinical management was at the discretion of the physician on duty. All patients who were diagnosed with AMI received antithrombotic treatment consisting of aspirin, a P2Y_12_ inhibitor, and low molecular weight heparin (LMWH), unless there was any contraindication. Stress testing was seldom performed in our hospital. In general, coronary computed tomography angiography (CCTA) was used to rule out ACS. Most low‐ and intermediate‐risk patients evaluated in the ED only received medical therapy. Patients with recurrent angina despite intensive medical therapy or with positive cTn detection were scheduled to undergo coronary angiography (CAG) unless they refused.

### 2.4 Data collection

Data were collected on standardized case report forms using clinical data standards by trained research staff.^14^ Both ED and hospital datasets were fully accessed. An electronic data capture system was used to guarantee the integrity and authenticity of the data. Patients were followed up by trained researchers at 30 days through telephone. The local death registry data were checked when patients were lost to follow up to ensure that they were deceased or not.

The outcomes included the LOS in the ED (time spent in the ED, covering time spent in the emergency observation room) at the index episode, hospital admission (admitted to an inpatient unit of Qilu Hospital of Shandong University or observed in the ED at least 24 hours) rate,^14^ the use of echocardiography, CCTA, CAG, and percutaneous coronary intervention (PCI), and the incidence of MACE at 30 days.

MACE included any of the following: all‐cause death, new or recurrent MI, emergency revascularization, stroke, cardiogenic shock, and cardiac arrest.

The final ED diagnoses and the MACE were adjudicated by two independent cardiologists. They reviewed all available medical records pertaining to the patient from the ED presentation to the 30‐day follow‐up. In situations of disagreement about the diagnoses or MACE, face‐to‐face meetings were called at regular intervals to reach a decision by consensus.

The final clinical diagnoses were divided into three categories: (a) ACS, including AMI and unstable angina (UA); (b) other cardiac diseases; (c) non‐cardiac diseases or diseases of unknown origin. AMI and UA were diagnosed according to the international recommendations, using the cTnI assay that was used for the patients in the clinical practice.^3^, ^14^ In brief, AMI referred to type 1 MI and was diagnosed when there was evidence of myocardial necrosis in a clinical setting consistent with myocardial ischemia due to definite or highly suspected plaque rupture and coronary thrombosis. Myocardial necrosis was identified by a detection of a rise and/or fall of cTnI with at least one value above the diagnostic threshold. According to the guideline, a minimum of a 20% cTn concentration change was required.^16^ UA was defined as angina occurring at rest and prolonged, or recent acceleration of angina increased from at least 1 Canadian Cardiovascular Society (CCS) class to at least CCS class III; it also included new‐onset angina of at least CCS class III, with coronary artery stenosis ≥70%, and without evidence of necrosis. Other cardiac diseases included type 2 MI (myocardial necrosis caused by arrhythmia, hypertensive urgency, coronary artery spasm, and the like),^3^ acute heart failure, myocarditis, pericarditis, and stable angina, among others.

### 2.5 Statistical analysis

Continuous variables were presented as means ± SDs or medians (with 25th and 75th percentiles), and ANOVA and Kruskal‐Wallis test were used to compare the differences when appropriate. Categorical variables were expressed as numbers and percentages. When comparing the differences, *x* ^2^ or Fisher's exact test were used.

Multivariable stepwise logistic regression analysis was used to investigate the association between the type of cTnI assay and the length of ED stay, hospital admission rate, the use of echocardiography, CCTA, CAG, PCI, and the incidence of MACE. Age, sex, previous MI, heart failure, hypertension, previous stroke, tobacco smoking, diabetes mellitus, family history, and ischemic ECG were included in the models.

All hypothesis testing was two‐tailed, and *P* values of less than 0.05 were considered to indicate statistical significance. Data were analyzed using SAS 9.4.

## 3 RESULTS

### 3.1 Clinical characteristics

**Figure 1:**
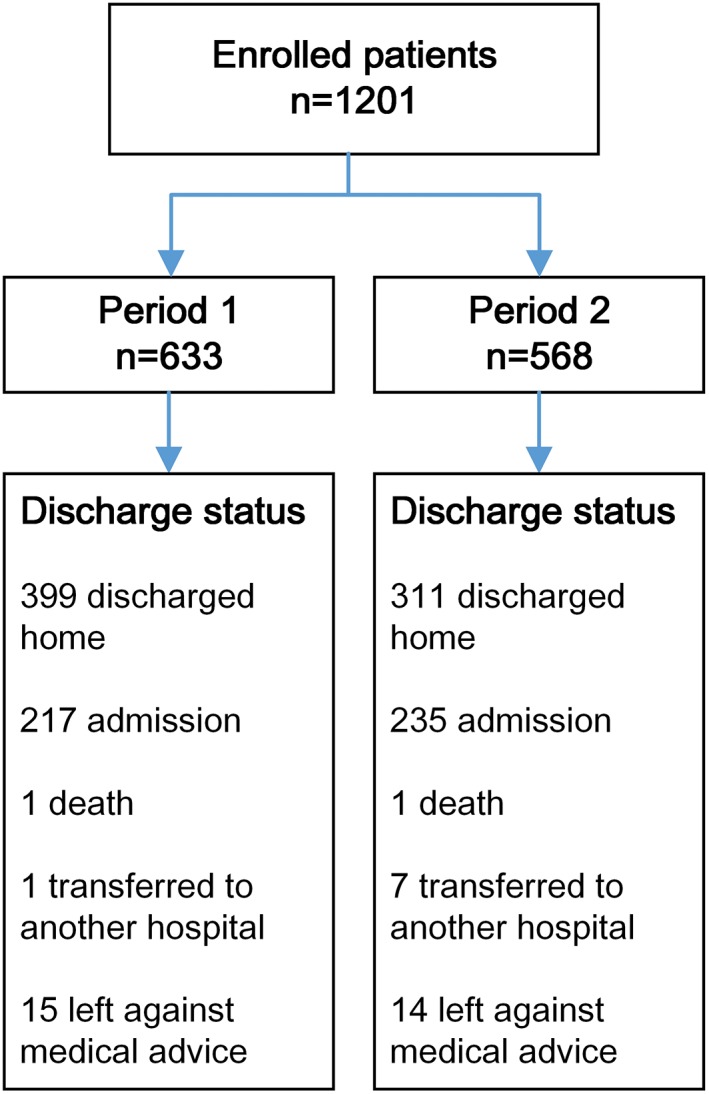
Flow diagram of patients in the study

A total of 1201 patients were identified for this study (study period 1, 633 visits; study period 2, 568 visits). Among them, 399 (63.03%) patients were discharged home in period 1 vs 311 (54.75%) patients in period 2 (Figure 1). The baseline characteristics of patients in these two groups were similar. Use of the more sensitive cTnI assay increased the proportion of patients with positive cTnI of the first measurement (10.90% vs 19.37%, *P* < 0.01), and decreased the proportion of patients with serial cTnI in the ED (15.01% vs 10.74%, *P* = 0.03), see Table 1.

**Table 1:** Clinical characteristics

	Period 1 (n = 633)	Period 2 (n = 568)	*P*‐value
Age, mean (SD), years	63.33 (13.39)	63.04 (13.17)	0.71
Male sex, n (%)	280 (44.23)	277 (48.77)	0.12
BMI, kg/m^2^, mean (SD)	25.19 (3.71)	25.12 (3.46)	0.74
Medical history, n (%)			
MI	112 (17.69)	110 (19.37)	0.46
Prior catheterization with stenosis ≥50%	146 (23.06)	150 (26.41)	0.18
PCI	116 (18.33)	115 (20.25)	0.40
CABG	8 (1.26)	15 (2.64)	0.08
Heart failure	10 (1.58)	12 (2.11)	0.49
Hypertension	363 (57.35)	331 (58.27)	0.74
Diabetes mellitus	143 (22.59)	125 (22.01)	0.81
Dyslipidemia	69 (10.90)	55 (9.68)	0.49
Stroke	82 (12.95)	70 (12.32)	0.74
chronic kidney disease	11 (1.74)	7 (1.23)	0.47
Peripheral artery disease	2 (0.32)	1 (0.18)	1.00
Smoker (current and past)	180 (28.44)	156 (27.46)	0.71
Family history, n (%)	134 (21.17)	96 (16.90)	0.06
ECG results, n (%)^a^			0.43
Ischemic	263 (43.33)	259 (47.87)	
Non‐diagnostic ST‐T changes	36 (5.93)	28 (5.18)	
Normal	225 (37.07)	180 (33.27)	
Other	83 (13.67)	74 (13.68)	
Clinical findings, mean (SD)			
Heart rate^b^ (beats per min)	80.07 (15.55)	79.28 (14.73)	0.37
Systolic blood pressure^c^ (mm Hg)	153.08 (24.90)	153.12 (24.42)	0.97
Diastolic blood pressure^c^ (mm Hg)	84.04 (14.73)	84.47 (15.90)	0.63
First cTnI > MI cut‐off, n (%)	69 (10.90)	110 (19.37)	<0.01
Serial cTnI in the ED, n (%)	95 (15.01)	61 (10.74)	0.03
Time interval to the second cTnI measurement, median (IQR), hour	5.90 (4.27, 8.60)	5.50 (3.97, 9.12)	0.46

Abbreviations: BMI, body mass index; CABG, coronary artery bypass grafting; cTnI, cardiac troponin I; ECG, electrocardiography; ED, emergency department; IQR, interquartile range; MI, myocardial infarction; PCI, percutaneous coronary intervention.

a Available data: n = 1138; period 1, n = 607; period 2, n = 531.

b Available data: n = 1197; period 1, n = 632; period 2, n = 565.

c Available data: n = 1191; period 1, n = 631; period 2, n = 560.

### 3.2 Final clinical diagnoses

Compared with period 1, more patients were considered to have AMI in period 2 (9.79% vs 16.02%, *P* < 0.01), while the proportion of patients with UA did not decrease significantly (23.38% vs 22.89%, *P* = 0.84) (Table 2).

**Table 2:** Final diagnoses during initial presentation

	Period 1 (n = 633)	Period 2 (n = 568)	*P*‐value
ACS, n (%)	210 (33.18)	221 (38.91)	0.04
AMI, n (%)	62 (9.79)	91 (16.02)	<0.01
UA, n (%)	148 (23.38)	130 (22.89)	0.84
Other cardiac disease, n (%)	56 (8.85)	49 (8.63)	0.89
Non‐cardiac or unknown disease, n (%)	367 (57.98)	298 (52.46)	0.06

Abbreviations: ACS, acute coronary syndrome; AMI, acute myocardial infarction; UA, unstable angina.

### 3.3 Medication in the ED, coronary status, and outcomes among patients who were discharged or hospitalized

Among patients who were discharged home or hospitalized, the proportions of patients who received aspirin, P2Y_12_ inhibitors, LMWH, and statins in the first 24 hours of care provided in the ED, and coronary status were not statistically different between these two periods. The median ED LOS of patients discharged or hospitalized did not decrease significantly (7.88 vs 6.99 hours, *P* = 0.24) after applying the more sensitive cTnI assay. For patients discharged home, the median ED LOS was shorter in period 2 (5.97 vs 2.32 hours, *P* < 0.01). The proportion of hospitalized patients increased when the more sensitive assay was used (35.23% vs 43.04%, *P* = 0.01) (Table 3).

**Table 3:** Medication in the first 24 hours in the ED, coronary status, and outcomes of patients discharged or hospitalized in the two groups

	Period 1 (n = 616)	Period 2 (n = 546)	*P*‐value
Medication in the first 24 hours in the ED, n (%)			
Aspirin	296 (48.05)	257 (47.07)	0.74
P2Y_12_ inhibitors	153 (24.84)	129 (23.63)	0.63
LMWH	111 (18.02)	81 (14.84)	0.14
Statins	150 (24.35)	149 (27.29)	0.25
Coronary status, n (%)			0.19
Normal/atheromatosis	23 (23.96)	29 (23.20)	
1 vessel disease	21 (21.88)	17 (13.60)	
2 vessel disease	24 (25.00)	27 (21.60)	
3 vessel disease	28 (29.17)	52 (41.60)	
ED LOS, median (IQR), hour			
All patients	7.88 (3.73, 14.64)	6.99 (1.97, 18.83)	0.24
Patients discharged from ED	5.97 (1.92, 8.98)	2.32 (1.63, 7.10)	<0.01
Patients hospitalized	21.67 (10.25, 44.82)	22.92 (10.83, 45.70)	0.71
Hospitalization, n (%)	217 (35.23)	235 (43.04)	0.01
Echocardiography, n (%)^a^	144 (23.38)	177 (32.42)	<0.01
CCTA, n (%)^a^	23 (3.73)	35 (6.41)	0.04
CAG, n (%)^a^	78 (12.66)	101 (18.50)	0.01
PCI, n (%)^a^	40 (6.75)	62 (15.23)	<0.01
MACE, n (%)^b^	17 (2.78)	9 (1.65)	0.20
Hospital readmission, n (%)^b^	67 (10.93)	38 (7.01)	0.02

Abbreviations: CAG, coronary angiography; CCTA, coronary computed tomography angiography; ED, emergency department; IQR, interquartile range; LMWH, low‐molecular‐weight heparin; LOS, length of stay; MACE, major adverse cardiac events; PCI, percutaneous coronary intervention.

a Data from emergency department and hospital.

b Available data: n = 1158; period 1, n = 612; period 2, n = 546.

Information for procedures are also provided in Table 3. More patients in period 2 received echocardiography (23.38% vs 32.42%, *P* < 0.01), CCTA (3.73% vs 6.41%, *P* = 0.04), CAG (12.66% vs 18.50%, *P* = 0.01), and PCI (6.75% vs 15.23%, *P* < 0.01).

In the follow‐up period, a total of 4 (0.63%) patients were lost in period 1, and 1 (0.18%) was lost in period 2. The incidences of MACE (2.78% vs 1.65%, *P* = 0.20) were not significantly different. The readmission rate decreased (10.93% vs 7.01%, *P* = 0.02) in period 2 (Table 3). Among patients directly discharged home in period 1, five patients suffered MACE, while no MACE occurred during period 2.

Following multivariable adjustment for potential confounders, the median LOS in the ED decreased in period 2 regarding patients discharged home (OR 0.39, 95% CI 0.28‐0.54, *P* < 0.01). The hospital admission rate (OR 1.43, 95% CI 1.12‐1.84, *P* < 0.01), the use of echocardiography (OR 1.58, 95% CI 1.22‐2.06, *P* < 0.01), CCTA (OR 1.78, 95% CI 1.04‐3.06, *P* = 0.04), CAG (OR 1.53, 95% CI 1.10‐2.12, *P* = 0.01) or PCI (OR 2.42, 95% CI 1.58‐3.70, *P* < 0.01) were increased, while the incidence of MACE did not change significantly (OR 0.61, 95% CI 0.27‐1.37, *P* = 0.23) (Table 4).

**Table 4:** Multivariable logistic regression, for length of ED stay, hospital admission rate, diagnostic procedures, treatments and MACE in relation to different cTnI assays for patients discharged or hospitalized

	Period 2 vs period 1 adjusted OR^a^ (95% CI)	*P*‐value
ED LOS of all patients > 7.48h^b^	0.78 (0.61, 1.00)	0.05
ED LOS of patients discharged from ED > 4.9h^c^	0.39 (0.28, 0.54)	<0.01
Hospitalization	1.43 (1.12, 1.84)	<0.01
Echocardiography	1.58 (1.22, 2.06)	<0.01
CCTA	1.78 (1.04, 3.06)	0.04
CAG	1.53 (1.10, 2.12)	0.01
PCI	2.42 (1.58, 3.70)	<0.01
MACE	0.61 (0.27, 1.37)	0.23

Abbreviations: CAG, coronary angiography; CCTA, coronary computed tomography angiography; ED, emergency department; LOS, length of stay; MACE, major adverse cardiac events; PCI, percutaneous coronary intervention.

a Confounding factors included age, sex, previous myocardial infarction, heart failure, hypertension, previous stroke, tobacco smoking, diabetes mellitus, prior catheterization with coronary stenosis ≥50%, family history, and ischemic electrocardiogram.

b 7.48 hours is the median LOS in the ED of all patients discharged home or hospitalized .

c 4.9 hours is the median LOS in the ED of patients discharged home in the study.

Among non‐ACS patients, no difference was found in the proportions of patients who underwent echocardiography, CCTA or CAG, and the incidence of MACE remained similar, too (Appendix table).

## 4 DISCUSSION

In this study, we evaluated the influence of a more sensitive cTnI assay on patient management and prognosis in actual medical practice. Several major findings were made.

First, we noted an increase in the proportion of patients diagnosed with AMI after introduction of the more sensitive cTnI assay. Second, the more sensitive cTnI assay was associated with a significant reduction of ED LOS in patients who were candidates for discharge. Third, the more sensitive cTnI assay significantly increased the hospital admission rate and the use of procedures. Fourth, the more sensitive cTnI assay did not correlate with a decrease in the incidence of MACE.

The clinical diagnostic threshold for the conventional cTnI assay during period 1 was the 10% CV level which was recommended by experts in the field.^17^ After the more sensitive cTnI assay was introduced, the 99th percentile URL was used as the MI cut‐off level. Lowering this cut‐off could lead to an increase in the rate of diagnosis of AMI.^18^ The proportion of patients diagnosed with AMI in our study was similar with that in Advantageous Predictors of Acute Coronary Syndrome Evaluation (APACE) trial.^13^ Usually, the implementation of cTn assays with increased sensitivity has contributed to a lower incidence of UA.^12^, ^13^, ^19^ However, the frequency of UA diagnose was not declined statistically in our study. The possible reason maybe that more patients with subtle increased cTn were diagnosed with UA in the absence of dynamic changes when using the more sensitive troponin. The overall ACS rate was higher in both pre‐ and post‐samples than the previous studies.^13^, ^20^ Different healthcare models might account for the distinction.

As found in the APACE trial, the introduction of a more sensitive assay was associated with shorter ED stays for patients discharged home.^13^ The rapid rule‐in or rule‐out protocols, such as 0/1 hour or 0/3 hours algorithms, were recommended for clinical use,^21^, ^22^ but had not been implemented in practice. Patients were dispositioned by unstructured clinical risk estimates, and the time interval to the second cTn measurement in the ED did not decrease significantly. However, due to the higher sensitivity allowing the early detection of AMI, clinician's confidence in ruling out or ruling in AMI had been improved, resulting in a more rapid exclusion or inclusion of AMI.

Our work showed that more patients were hospitalized and more procedures were performed after the more sensitive assay was implemented, partly because of the growing proportion of patients diagnosed with ACS. The procedures for patients with non‐ACS did not increase. The changes of procedures after the introduction of more sensitive assays in patients with acute chest pain presenting to the ED were inconclusive in previous studies.^11^, ^12^, ^13^ Yip et al found the use of CAG was increased, while revascularization procedures did not alter statistically.^11^ Sanchis et al showed that use of the high‐sensitivity assay increased the rates of CAG and revascularization, while led to a reduction of non‐invasive tests.^12^ However, no increase of invasive procedures was found in the APACE trial.^13^ Eggers et al found that an improved sensitivity cTn assay increased the number of patients diagnosed with ACS and identified more patients suitable for diagnostic procedures for those admitted to coronary care units in SWEDEHEART registry.^23^ The management strategies and the inclusion criteria of different studies may lead to the inconsistency of the results. The approaches to management acute chest pain differed among countries and centers. Stress tests were seldom performed in our hospital. Rate of patients referred for CAG was lower than those in the SNAPSHOT ACS registry and the APACE trial.^1^, ^13^ Rates of patients referred for echocardiography and CCTA were close to those in the SNAPSHOT ACS registry. The variation of selection criteria for patients undergoing troponin testing markedly influenced the positive predictive value for a diagnosis of AMI,^24^ and in addition, it affected clinical management.

The use of the more sensitive cTn assay did not result in fewer adverse events. Although Mills and his team found that lowering the diagnostic threshold for AMI with a sensitive cTn assay could improve clinical outcomes,^18^ several observational studies stated that no change in the incidence of MACE was found, with or without an increase in revascularizations.^11^, ^12^ High‐STEACS, a stepped‐wedge, cluster‐randomized controlled trial also showed that the use of a high‐sensitivity cTn was not associated with a lower incidence of MACE.^6^ Troponin cannot be a substitute for careful clinical assessment, and it must be interpreted in the context of the history of each patient. More researches are necessary to determine the preferred clinical protocols to integrate the high‐sensitivity assays into the clinical practice.

Our study had several limitations. First, it was a single center observational study and the sample scale was small. Different healthcare models and potential confounding factors that were unmeasured may influence the results. The study needs to be repeated on larger scales and with multi‐center trials. Second, we evaluated two cTnI assays, which had different analytical performances and were released by the same supplier. The results may not be generalized to other cTn assays. Finally, we did not collect data on coronary artery bypass grafting and discharge medication, and the follow‐up period in our study was short. The long‐term outcomes should be further researched.

## 5 CONCLUSIONS

The implementation of the more sensitive cTn assay was likely to identify patients for safe discharge much earlier, although prompted more hospital admissions and procedures without impacting on the incidence of MACE at 30 days.

## CONFLICT OF INTEREST

The authors declare no potential conflict of interests.

## Supporting information


**Appendix S1.** Table Procedures and MACE of patients discharged or hospitalized with non‐ACS in the two groups

## References

[1] Cullen L , French JK , Briffa TG , et al. Availability of highly sensitive troponin assays and acute coronary syndrome care: insights from the SNAPSHOT registry. Med J Aust. 2015;202(1):36‐39. 25588444 10.5694/mja13.00275

[2] Reichlin T , Twerenbold R , Reiter M , et al. Introduction of high‐sensitivity troponin assays: impact on myocardial infarction incidence and prognosis. Am J Med. 2012;125(12):1205‐1213. 23164485 10.1016/j.amjmed.2012.07.015

[3] Thygesen K , Alpert JS , Jaffe AS , et al. Third universal definition of myocardial infarction. Circulation. 2012;126(16):2020‐2035. 22923432 10.1161/CIR.0b013e31826e1058

[4] Reichlin T , Hochholzer W , Bassetti S , et al. Early diagnosis of myocardial infarction with sensitive cardiac troponin assays. N Engl J Med. 2009;361(9):858‐867. 19710484 10.1056/NEJMoa0900428

[5] Bonaca MP , Ruff CT , Kosowsky J , et al. Evaluation of the diagnostic performance of current and next‐generation assays for cardiac troponin I in the BWH‐TIMI ED chest pain study. Eur Heart J Acute Cardiovasc Care. 2013;2(3):195‐202. 24222830 10.1177/2048872613486249 PMC3821818

[6] Shah ASV , Anand A , Strachan FE , et al. High‐sensitivity troponin in the evaluation of patients with suspected acute coronary syndrome: a stepped‐wedge, cluster‐randomised controlled trial. Lancet. 2018;392(10151):919‐928. 30170853 10.1016/S0140-6736(18)31923-8 PMC6137538

[7] Sanchis J , Abellan L , Garcia‐Blas S , et al. Usefulness of delta troponin for diagnosis and prognosis assessment of non‐ST‐segment elevation acute chest pain. Europ Heart J Acute Cardiovasc Care. 2016;5(5):399‐406. 10.1177/2048872615593534 26136512

[8] Bjurman C , Larsson M , Johanson P , et al. Small changes in troponin T levels are common in patients with non‐ST‐segment elevation myocardial infarction and are linked to higher mortality. J Am Coll Cardiol. 2013;62(14):1231‐1238. 23933541 10.1016/j.jacc.2013.06.050

[9] Morrow DA , Bonaca MP . Real‐world application of "delta" troponin diagnostic and prognostic implications. J Am Coll Cardiol. 2013;62(14):1239‐1241. 23933544 10.1016/j.jacc.2013.06.049

[10] Papendick C , Blyth A , Seshadri A , et al. A randomized trial of a 1‐hour troponin T protocol in suspected acute coronary syndromes: Design of the Rapid Assessment of possible ACS in the emergency department with high sensitivity troponin T (RAPID‐TnT) study. Am Heart J. 2017;190:25‐33. 28760210 10.1016/j.ahj.2017.05.004

[11] Yip TPY , Pascoe HM , Lane SE . Impact of high‐sensitivity cardiac troponin I assays on patients presenting to an emergency department with suspected acute coronary syndrome. Med J Aust. 2014;201(3):158‐161. 25128951 10.5694/mja13.00117

[12] Sanchis J , Garcia‐Blas S , Mainar L , et al. High‐sensitivity versus conventional troponin for management and prognosis assessment of patients with acute chest pain. Heart. 2014;100(20):1591‐1596. 24947318 10.1136/heartjnl-2013-305440

[13] Twerenbold R , Jaeger C , Rubini Gimenez M , et al. Impact of high‐sensitivity cardiac troponin on use of coronary angiography, cardiac stress testing, and time to discharge in suspected acute myocardial infarction. Eur Heart J. 2016;37(44):3324‐3332. 27357358 10.1093/eurheartj/ehw232 PMC5177796

[14] Cannon CP , Brindis RG , Chaitman BR , et al. ACCF/AHA key data elements and definitions for measuring the clinical management and outcomes of patients with acute coronary syndromes and coronary artery disease: a report of the American College of Cardiology Foundation/American Heart Association Task Force on Clinical Data Standards (Writing Committee to Develop Acute Coronary Syndromes and Coronary Artery Disease Clinical Data Standards). Circulation. 2013;127(9):1052‐1089. 23357718 10.1161/CIR.0b013e3182831a11

[15] Uettwiller‐Geiger D , Wu AH , Apple FS , et al. Multicenter evaluation of an automated assay for troponin I. Clin Chem. 2002;48(6 Pt 1):869‐876. 12029002

[16] Amsterdam EA , Wenger NK , Brindis RG , et al. 2014 AHA/ACC guideline for the management of patients with non‐ST‐elevation acute coronary syndromes: a report of the American College of Cardiology/American Heart Association Task Force on Practice Guidelines.Circulation. 2014;130(25):e344‐e426. 25249585 10.1161/CIR.0000000000000134

[17] Apple FS , Wu AHB , Jaffe AS . European Society of Cardiology and American College of cardiology guidelines for redefinition of myocardial infarction: how to use existing assays clinically and for clinical trials. Am Heart J. 2002;144(6):981‐986. 12486421 10.1067/mhj.2002.124048

[18] Mills NL , Churchhouse AM , Lee KK , et al. Implementation of a sensitive troponin I assay and risk of recurrent myocardial infarction and death in patients with suspected acute coronary syndrome. JAMA. 2011;305(12):1210‐1216. 21427373 10.1001/jama.2011.338

[19] Sandoval Y , Apple FS , Smith SW . High‐sensitivity cardiac troponin assays and unstable angina. Eur Heart J Acute Cardiovasc Care. 2018;7(2):120‐128. 27388716 10.1177/2048872616658591

[20] Christenson J , Innes G , McKnight D , et al. A clinical prediction rule for early discharge of patients with chest pain. Ann Emerg Med. 2006;47(1):1‐10. 16387209 10.1016/j.annemergmed.2005.08.007

[21] Mueller C . Biomarkers and acute coronary syndromes: an update. Eur Heart J. 2014;35(9):552‐556. 24357507 10.1093/eurheartj/eht530

[22] Roffi M , Patrono C , Collet JP , et al. 2015 ESC guidelines for the management of acute coronary syndromes in patients presenting without persistent ST‐segment elevation: task force for the Management of Acute Coronary Syndromes in patients presenting without persistent ST‐segment elevation of the European Society of Cardiology (ESC). Eur Heart J. 2016;37(3):267‐315. 26320110 10.1093/eurheartj/ehv320

[23] Eggers KM , Lindahl B , Melki D , Jernberg T . Consequences of implementing a cardiac troponin assay with improved sensitivity at Swedish coronary care units: an analysis from the SWEDEHEART registry. Eur Heart J. 2016;37(30):2417‐2424. 26916797 10.1093/eurheartj/ehw029

[24] Shah ASV , Sandoval Y , Noaman A , et al. Patient selection for high sensitivity cardiac troponin testing and diagnosis of myocardial infarction: prospective cohort study. BMJ. 2017;359:j4788. 29114078 10.1136/bmj.j4788 PMC5683043

