# Supplementary material for: Clinical impact of using a more sensitive troponin assay in patients with acute chest pain
Source: Clin Cardiol. 2019 Mar 29;42(5):561–7. doi: 10.1002/clc.23177 (PMC6522991; doi:10.1002/clc.23177)
Supplement: Supplementary file 1 — Appendix S1. Table Procedures and MACE of patients discharged or hospitalized with non‐ACS in the two groups [file CLC-42-561-s001.docx]

**Appendix Table Procedures and MACE of patients discharged or hospitalized with** **non-ACS in the two groups**

|  | **Period 1 (n=423)** | **Period 2 (n=347)** | **p-value** |  |
| --- | --- | --- | --- | --- |
| **Echocardiography, n (%)^a^** | 51 (12.06) | 49 (14.12) | 0.40 |  |
| **CCTA, n (%)^a^** | 15 (3.55) | 17 (4.90) | 0.35 |  |
| **CAG, n (%)^a^** | 11 (2.60) | 9 (2.59) | 1.00 |  |
| **MACE, n (%)** | 7 (1.67) | 1 (0.29) | 0.08 |  |

^a^ Data from emergency department and hospital.

ACS, acute coronary syndrome; CAG, coronary angiography; CCTA, coronary computed tomography angiography; MACE, major adverse cardiac events.
